# A rare case of fenpyroximate poisoning with presenting anticholinesterase poisoning signs

**DOI:** 10.1002/ccr3.3159

**Published:** 2020-07-20

**Authors:** Soodabeh Kheirkhah, Kiumars Bahmani

**Affiliations:** ^1^ Department of Internal Medicine Minab Abolfazl Hospital Hormozgan University of Medical Science Hormozgan Iran; ^2^ Department of Pharmacology and Toxicology School of Pharmacy Shahid Beheshti University of Medical Science Tehran Iran

**Keywords:** anticholinesterase, fenpyroximate, poisoning

## Abstract

We report a case of fenpyroximate poisoning with cholinergic signs that could be due to mixing it with anticholinesterase. Clinicians should consider co‐ingestion of pesticides to proper diagnosis and management of the pesticide poisoning.

## Graphical abstract

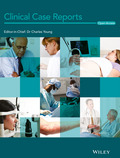

## 1 INTRODUCTION

Fenpyroximate is a mitochondrial proton‐translocating NADH ubiquinone oxidoreductase (complex I) inhibitor.^1^ It is used in the agricultural sector as an acaricide. Acute and chronic toxicity studies of fenpyroximate in laboratory animals have been conducted by oral lavage.^2^ There are few case reports of poisoning with fenpyroxymate, and there is no report of fenpyroxymate poisoning with co‐ingestion of other drugs and poisons. In this study, we report a rare case of fenpyroximate poisoning with unusual symptoms suspected with anticholinesterase co‐ingestion.

## 2 CASE PRESENTATION

A 29‐year‐old man admitted to the hospital following ingestion of 15 tablets of alprazolam 0.5 mg and 15 tablets of valproate sodium 500 mg and 120 mL of fenpyroximate 5%. The patient was under medical care with alprazolam and valproate. At the presentation, the patient was unconscious, with sialorrhea, lacrimation, and diarrhea; emesis, sweating, and pulmonary; and upper airway secretions, but he had not miosis and pupils were normal in size. His systolic blood pressure and heart rate were 60 mm Hg and 120, respectively. Venous blood gas analysis showed metabolic acidosis (pH 7.35; PCO2 42 mm Hg; PO2 62.1 mm Hg; base excess −3 mmol/L,). Cardiac function was normal, EKG and echocardiography were performed, and ejection fraction was %50. We performed gastric lavage and admit the patient in the intensive care unit. Atropine challenge test was positive; therefore, co‐ingestion of an anticholinesterase agent (organophosphate or carbamate) suspected, but butyrylcholinesterase activity measuring was not available at the time for confirmation so atropine was administrated considering cholinergic signs of the patient. After 14 hours, cholinergic signs diminished and atropine administration was discontinued. According to anesthetist consultation, the patient was not intubated, one day after admission, patient's body temperature raised, leukocytosis has been documented with domination of neutrophils, and ceftriaxone and clindamycin were administrated for him until discharging from ICU. The patient was discharged after 9 days hospitalization with clinically stable conditions and referred to psychiatrist for the further treatment. Although the patient was discharged in good general condition, but due to pyrosis, endoscopic gastric evaluation was performed at the follow‐up, which was reported to be erosive gastropathy.

## 3 DISCUSSION

Fenpyroximate is an acaricide that inhibit complex I of the mitochondrial respiratory chain.^3^ They decrease ATP production and therefore ATP content of the cell.^4^ The symptoms and signs of toxicity include the following: nausea, vomiting, incoordination, convulsions, central nervous system depression and respiratory distress, bradycardia, and arrhythmia.^5^ However, in this patient remarkably has shown with signs and symptoms of anticholinesterase poisoning that could be on the consequence of co‐ingestion with most likely organophosphate or carbamate pesticide. This patient had not miosis, one of the most frequent findings in anticholinesterase poisoning; however, it could be due to co‐ingestion of alprazolam and valproate.^6^, ^7^ Jallow MF et al research showed that the farmers were not aware of safety measures of pesticide using^8^ consequently most of them mixes the pesticides.^9^ Lee HY et.al reported severe metabolic acidosis following ingestion of 70ml of fenpyroximate ^10^; however, in the present patient metabolic acidosis was mild that might be because of immediate referring to hospital and decontamination. Kuruppu Arachchi et al. reported acute neurotoxicity as consequence of fenpyroximate poisoning,^1^ but our patient had not sign of acute neurotoxicity. The inhibition of mitochondrial complex I leading to dysfunction of mitochondria and generation of reactive oxygen species is hypothesized as a mechanism of fenpyroximate induced neurotoxicity,^11^ and there is a case report of a 58‐year‐old man who developed parkinsonism two years after intentional fenpyroximate intoxication.^12^ Lack of acute neurotoxicity in our patient could be result of alprazolam co‐ingestion because benzodiazepines have neuroprotective effects. They enhance GABA transmission and thus counteract excitotoxic degenerative mechanisms and also reduce and cerebral metabolic rate.^13^

## 4 CONCLUSION

Pesticide poisoning can be caused by mixing pesticides with each other or other drugs and poisons sometimes. They can cause misdiagnosis and increase the severity of complications, or they might have protective effects like alprazolam in mentioned case. Our case had mix signs and symptoms of fenpyroximate and anticholinesterase poisoning, and clinicians should be aware of this for proper diagnosis and treatment of pesticide poisoning cases.

## CONFLICT OF INTEREST

None declared.

## AUTHOR CONTRIBUTIONS

Dr. Soodabeh Kheirkhah: was responsible for visiting the patient, physical examination, management, follow‐up of the patient, and reviewing the manuscript. Dr. Kiumars Bahmani: was responsible for toxicology consultation, drafting the manuscript, and revision of the manuscript.
